# Development of an ensemble Kalman filter data assimilation system for the Venusian atmosphere

**DOI:** 10.1038/s41598-017-09461-1

**Published:** 2017-08-24

**Authors:** Norihiko Sugimoto, Akira Yamazaki, Toru Kouyama, Hiroki Kashimura, Takeshi Enomoto, Masahiro Takagi

**Affiliations:** 1 Research and Educatin Center for Natural Sciences/Department of Physics, Keio University, Yokohama, 223-8521 Japan; 2 Application Laboratory, Japan Agency for Marine-Earth Science and Technology, Yokohama, 236-0001 Japan; 3 Information Technology Research Institute, National Institute of Advanced Industrial Science and Technology, Tsukuba, 305-8568 Japan; 4 Center for Planetary Science/Department of Planetology, Kobe University, Kobe, 650-0047 Japan; 5 Disaster Prevention Research Institute, Kyoto University, Uji, 611-0011 Japan; 6 Faculty of Science, Kyoto Sangyo University, Kyoto, 603-8555 Japan

## Abstract

The size and mass of Venus is similar to those of the Earth; however, its atmospheric dynamics are considerably different and they are poorly understood due to limited observations and computational difficulties. Here, we developed a data assimilation system based on the local ensemble transform Kalman filter (LETKF) for a Venusian Atmospheric GCM for the Earth Simulator (VAFES), to make full use of the observational data. To examine the validity of the system, two datasets were assimilated separately into the VAFES forecasts forced with solar heating that excludes the diurnal component Qz; one was created from a VAFES run forced with solar heating that includes the diurnal component Qt, whereas the other was based on observations made by the Venus Monitoring Camera (VMC) onboard the Venus Express. The VAFES-LETKF system rapidly reduced the errors between the analysis and forecasts. In addition, the VAFES-LETKF system successfully reproduced the thermal tide excited by the diurnal component of solar heating, even though the second datasets only included horizontal winds at a single altitude on the dayside with a long interval of approximately one Earth day. This advanced system could be useful in the analysis of future datasets from the Venus Climate Orbiter ‘Akatsuki’.

## Introduction

During the past two decades, data assimilation has become an effective tool in planetary atmospheric science. Observation data are sampled irregularly in space and time, even in studies of the Earth’s atmosphere. Therefore, the global and continuous datasets produced by general circulation models (GCMs) that use data assimilation are considerably useful tools because they are dynamically and thermodynamically consistent. Amongst several data assimilation schemes, the local ensemble transform Kalman filter (LETKF)^1^ is one of the most powerful and efficient schemes. Hence, it has been successfully applied to the terrestrial^2, 3^ and Martian^4, 5^ atmospheres.

Data assimilation has not yet been attempted for the Venusian atmosphere. This may not only be attributed to the limited amount of meteorological observations available but also to the computational difficulties arising from employing theoretical models and GCMs. Hence, a realistic structure of the Venusian atmosphere has not been produced using GCMs. Recently, we developed a Venusian atmospheric GCM, named AFES-Venus (referred to as VAFES in this paper), based on the Atmospheric GCM for the Earth Simulator (AFES)^6^. Because AFES is highly optimized for the Earth Simulator, one of the world’s largest vector super-computers provided by the Japan Agency for Marine-Earth Science and Technology (JAMSTEC), one advantage of the VAFES is that we can perform high-resolution simulations to reproduce realistic structures of the Venusian atmosphere. Using the VAFES, we successfully investigated barotropic/baroclinic instability waves^7, 8^ and elucidated a puzzling temperature structure, called the ‘cold collar’, at high latitudes^9^. Though the VAFES is a simple dynamical model wherein the distribution of solar heating is prescribed, a Newtonian cooling scheme is used to simplify the infrared radiative transfer and the cloud physics is not included, the atmospheric structures reproduced by the model were in good agreement with the observations. Therefore, it is expected that the VAFES can be used for data assimilation. The AFES-LETKF data assimilation system has already been developed^2^ for the terrestrial atmosphere, and the experimental ensemble reanalysis^10–13^ has been successfully performed.

The VMC^14^ provided Venusian cloud images at the cloud-top level (approximately 70 km) for over eight years since April 2006. From these images, we derived daily horizontal winds at approximately 70 km using cloud tracking for a period from May 2006 to January 2010^15^. We used these wind data for data assimilation in a test case, although the data were spatiotemporally sparse as compared to the terrestrial observational data.

In this study, we developed the VAFES-LETKF system wherein the LETKF was applied to the VAFES, and it was tested with two observational datasets. This was the first data assimilation experiment for the Venusian atmosphere. The two datasets included horizontal winds at only a single altitude above the cloud top. One dataset was synthesized observational data produced by a VAFES simulation run forced by Qt (solar heating that includes the diurnal component), whereas the other dataset was observational data based on VMC^15^. The VAFES forecasts, which were assimilated with the observational data, were produced from a VAFES simulation run forced by Qz (solar heating that excludes the diurnal component). This indicated that the thermal tide, which is strongly excited in the cloud layer by the diurnal component of solar heating^16–18^ and significantly affects the superrotation^19, 20^, was not included in the basic run. Therefore, if the VAFES-LETKF system functions appropriately, it is anticipated that the thermal tide would be reproduced in the runs using both observational datasets. Our primary goal was to demonstrate that the VAFES-LETKF data assimilation system functions appropriately, which can be useful for future observations.

## Results

### Effect of data assimilation

In a data assimilation scheme, an improved estimate (referred to as an analysis) is derived by combining observations and short-time forecasts. The LETKF is a type of the ensemble square root Kalman filter that seeks the analysis solution with minimum error variance. Using a 31-member ensemble of VAFES simulation runs, the uncertainty of the model forecast was characterized in the current system. The minimal interval for the data assimilation cycle was 6 h. The four-dimensional LETKF uses 7-h time slots to produce each analysis. Therefore, observations can be assimilated every hour if they are available^1, 21^.

Tables 1 and 2 provide a summary of our experiments. We prepared several idealised observations of 70-km horizontal winds for the cloud-top level at intervals of 1, 6 and 24 h (Cases H1, H6 and H24, respectively). These idealised observations were produced by the VAFES simulation run forced by Qt (Case Qt wherein solar heating excites the thermal tide). In accordance with real satellite observations, we used single-altitude horizontal winds. The other observational data were based on cloud tracking of the ultraviolet images captured by the VMC^15^ (Case Vmc), which included approximately 70-km horizontal winds located in a narrow dayside region from approximately 60°S to 30°N between approximately 07:00 local time (LT) and 17:00 LT, which correspond to 80°W and 80°E longitudes, provided the sub-solar point (12:00 LT) was positioned at 0°E longitude. Note that the sub-solar point was set to move westwards, which was consistent with the direction of the planet rotation assumed in the VAFES. The time intervals of the VMC horizontal wind data were approximately one Earth day. In this study, we used 73 observations of horizontal winds during a period from 28 January 2008 to 26 April 2008. All observations captured the thermal tide component; however, the VAFES forecasts to be assimilated did not capture this component because the diurnal component of solar heating was excluded (Case Qz wherein solar heating only included the zonal mean component). This indicates that atmospheric motions in all test cases would ‘relax’ to those in Case Qz when there was no observation. In addition, a free-run forecast (Case Frf) was performed to produce a background that employed a 31-ensemble of Case Qz runs without observations, i.e. without data assimilation. In all runs, the resolution was fixed to T42L60, with 128 times 64 grids and 60 layers extending horizontally and vertically, respectively, from the flat ground to 120 km. To set up the experiments, we performed numerical integrations from an idealised superrotating state for Cases Qz and Qt for four Earth years. The modelled atmospheres reached quasi-steady states within approximately one Earth year and were maintained for more than 10 Earth years^8^. The results for Cases H1, H6, H24, Vmc and Frf comprise a 31-ensemble mean of each member.

**Table 1:** Free Venusian Atmospheric GCM for the Earth Simulator (VAFES) control runs of Cases Qz and Qt.

Cases	Solar heating
Qz	Zonal mean component only
Qt	Including diurnal variation

Case Qz is forced with solar heating that includes zonal mean component only, while Case Qt is forced with solar heating that includes the diurnal variation. The thermal tide is directly excited by solar heating in Case Qt but not in Case Qz. Case Qt is used to generate the idealised observations.

**Table 2:** Assimilation runs of Cases H1, H6, H24 and Vmc and free run forecast of Case Frf.

Cases	Observations	Intervals	VAFES
H1	Qt	1 h	Qz
H6	Qt	6 h	Qz
H24	Qt	24 h	Qz
Vmc	VMC	Approximately 24 h	Qz
Frf	None	None	Qz

Idealised observational data for Cases H1, H6 and H24 are prepared by the simulation run of Case Qt with different intervals: 1, 6 and 24 h. Real observational data for Case Vmc is based on the ultraviolet (UV) images captured by the Venus Monitoring Camera (VMC)^15^ with approximately one Earth day interval. All cases are forced with solar heating, including only the zonal mean component (which is the same setting as Case Qz), i.e. the thermal tide is excluded.

**Figure 1:**
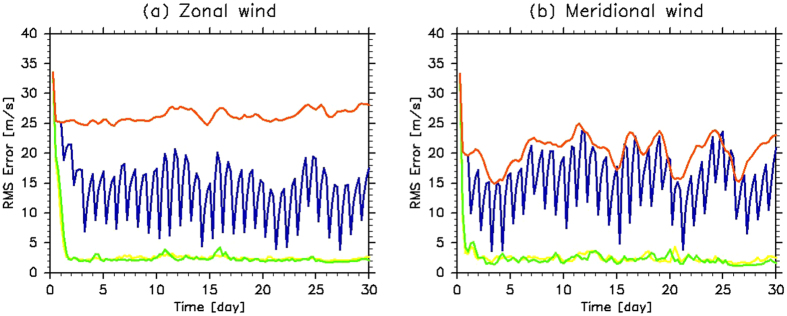
Time evolutions of the root-mean-square (RMS) errors from idealised observations at 70 km in (**a**) zonal and (**b**) meridional winds (m s^−1^) for Cases H1 (1-h observations; yellow), H6 (6-h observations; green), H24 (24-h observations; blue) and Frf (background; red).

**Figure 2:**
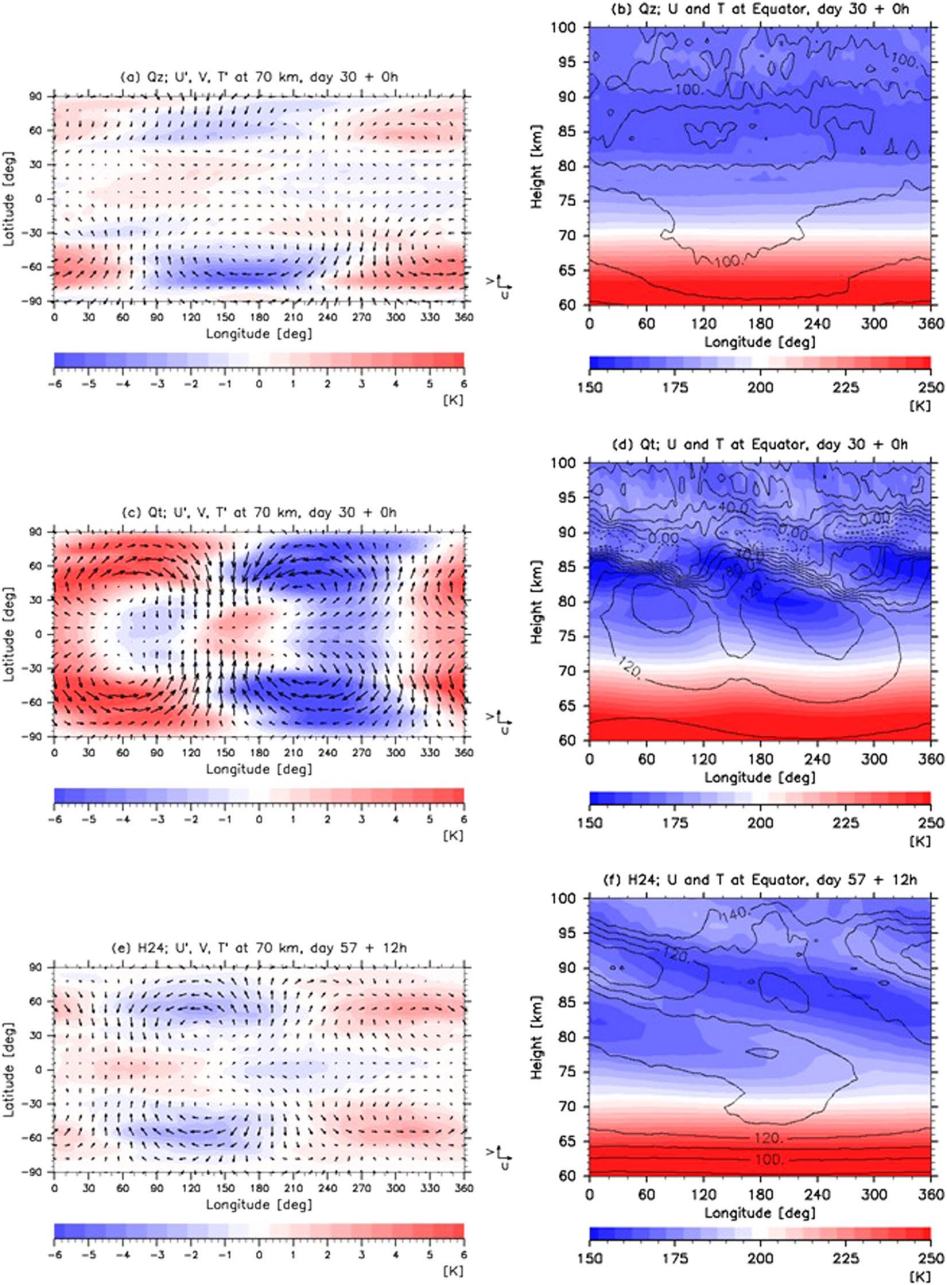
Horizontal and vertical distributions of temperature (T; colour shades; K) for Cases (**a**,**b**) Qz, (**c**,**d**) Qt at day 30 midnight and (**e**,**f**) H24 at day 57 noon. Prime indicates the disturbance from its zonal mean. The slowly varying components are extracted using a low-pass filter (applying running mean) with a cut-off period longer than four Earth days. In panels (**a**,**c** and **e**), horizontal distributions of horizontal winds ‘U’ (zonal) and ‘V’ (meridional) at 70 km are depicted (black vectors; unit: 25 m s^−1^). In panels (**b**,**d** and **f**), vertical distributions of zonal wind at the equator are depicted (black contours; intervals are 20 m s^−1^ for (**d**) and 10 m s^−1^ for (**b** and **f**), respectively). Cases Qt and Qz are the Venusian Atmospheric GCM for the Earth Simulator (VAFES) runs with and without the diurnal component of solar heating, respectively. Note that the thermal tide is directly excited by solar heating in Case Qt but not in Case Qz.

**Figure 3:**
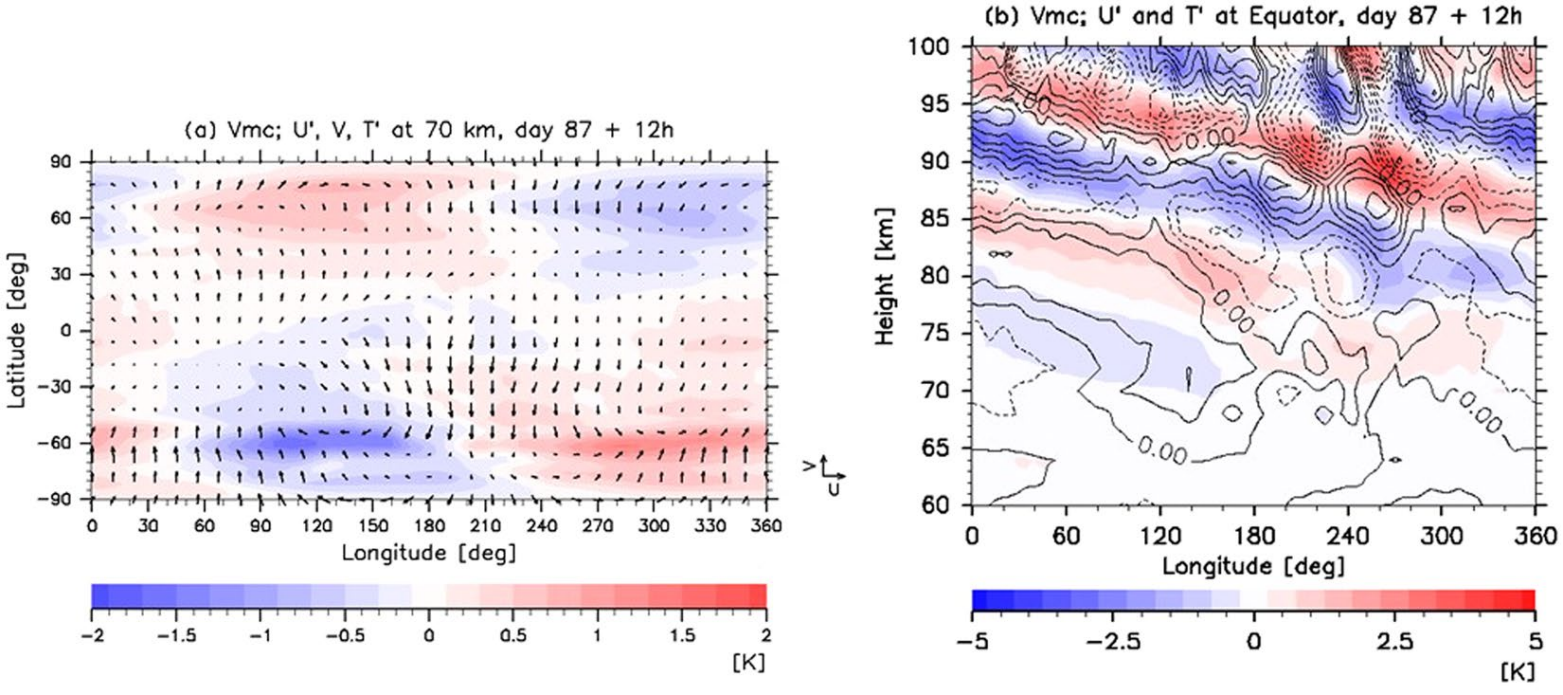
(**a**) Horizontal and (**b**) vertical distributions of temperature deviation ‘${\bf{T}}^{\prime}$’ obtained from zonally averaged temperature (colour shades; K) associated with the thermal tide for Case Vmc at day 87 midnight noon. Prime indicates disturbance from its zonal mean. In Case Vmc, real observations based on the Venus Monitoring Camera (VMC) onboard the Venus Express are assimilated. The slowly varying components (thermal tide) are extracted using a low-pass filter with a cut-off period longer than four Earth days. In panel (**a**), a horizontal distribution of horizontal winds ‘U’ (zonal) and ‘V’ (meridional) at 70 km is also depicted (black vectors; unit: 25 m s^−1^; zonal wind is a deviation from its zonal mean). In panel (**b**), a vertical distribution of zonal wind deviation ‘${\rm{U}}^{\prime}$’ from its zonal mean at the equator is depicted (black contours; intervals are 1 m s^−1^).

Figure 1 shows that the VAFES-LETKF data assimilation system rapidly reduced the root-mean-square (RMS) error between the analysis and the subsequent forecast at each grid point at 70 km for both the zonal and meridional winds, except for Case Frf (background). For Case H24, the cycle of data assimilation (i.e. executed once daily), was clearly apparent; however, the RMS error was smaller than that for Case Frf which did not include observations. While the meridional winds were by an order of magnitude smaller than the zonal winds, the meridional winds associated with disturbances are of the order same as that of the zonal winds with disturbances. This is why the RMS errors for the zonal and meridional winds during assimilation were almost of the same order. Note that the RMS error in the temperature field did not converge, even for Case H1 (not shown), although the temperature was also modified to be in balance with horizontal winds (Figs 2 and 3). Because the temperature field balanced with horizontal winds can be produced such that its horizontal average remains unchanged, the difference in the reference temperatures of Cases Qz and Qt (approximately a few degrees K) did not converge with data assimilation conducted using only horizontal winds. It was confirmed that the RMS error of the temperature field converged when the temperature was included in the observational data (not shown).

### Reproducibility of the thermal tide

The thermal tide is a global-scale atmospheric wave excited by solar heating, which moves along with the Sun. Because approximately 60% of the solar flux is absorbed at the cloud levels of 45–70 km on Venus, the thermal tide is strongly excited in this location, and it propagates vertically. It has been expected from theoretical researches that components with the zonal wave numbers of 1 and 2, referred to as the diurnal and semidiurnal tides, respectively, would be predominant at the cloud levels^16, 17^. Below (Above) the cloud-top level, the amplitude of the diurnal (semidiurnal) tide was larger than that of the semidiurnal (diurnal) tide. These structures of the thermal tide can be observed in Case Qt (Fig. 2c and d) wherein the thermal tide was directly excited by solar heating. The westward phase tilt with height indicates the upward propagation of the thermal tide.

Even though the observational data were corrected only for the 70-km winds, Fig. 2e and f show that the three-dimensional structure associated with the thermal tide appears clearly, even in Case H24, and it propagates upwards above 70 km. Note that the RMS errors for the zonal and meridional winds in Case H24 do not converge. These errors were considerably reduced only when the observations were conducted. Nevertheless, the thermal tide structure with a zonal wave number of 1, which is similar to that obtained for Case Qt (Fig. 2c and d), was found in the temperature field, even though the temperature was not included in the observational data. Compared with Case Qt, the amplitude of the thermal tide found in Case H24 was approximately half of that found in Case Qt. It is worth noting that the VAFES-LETKF data assimilation system successfully reproduced the thermal tide not only in the horizontal winds but also in the temperature field. This was done by assimilating the temporally sparse observational data that did not include the temperature (once a day for Case H24). The thermal tide and its vertical propagation were not present for Case Qz (Fig. 2a and b) wherein the thermal tide was eliminated by excluding the diurnal component from solar heating. These results clearly showed that the data assimilation with the inclusion of the thermal tide component in the horizontal winds produced temperature deviations associated with the thermal tide as a dynamically balanced state. Since the vertical shear of the zonal mean zonal wind was different between Cases H24 and Qt, the inclinations of the phase of the thermal tide differed from each other.

Figure 3 shows the results for Case Vmc. The thermal tide was successfully reproduced, even though only 70-km-altitude horizontal winds were used. These winds were located on the dayside of the southern hemisphere with a time interval of approximately 24 h. The wind and temperature components antisymmetric about the equator were also induced by the meridional winds. These winds move across the equator and were obtained from the VMC data^15^.

### Impact of data assimilation on general circulation

**Figure 4:**
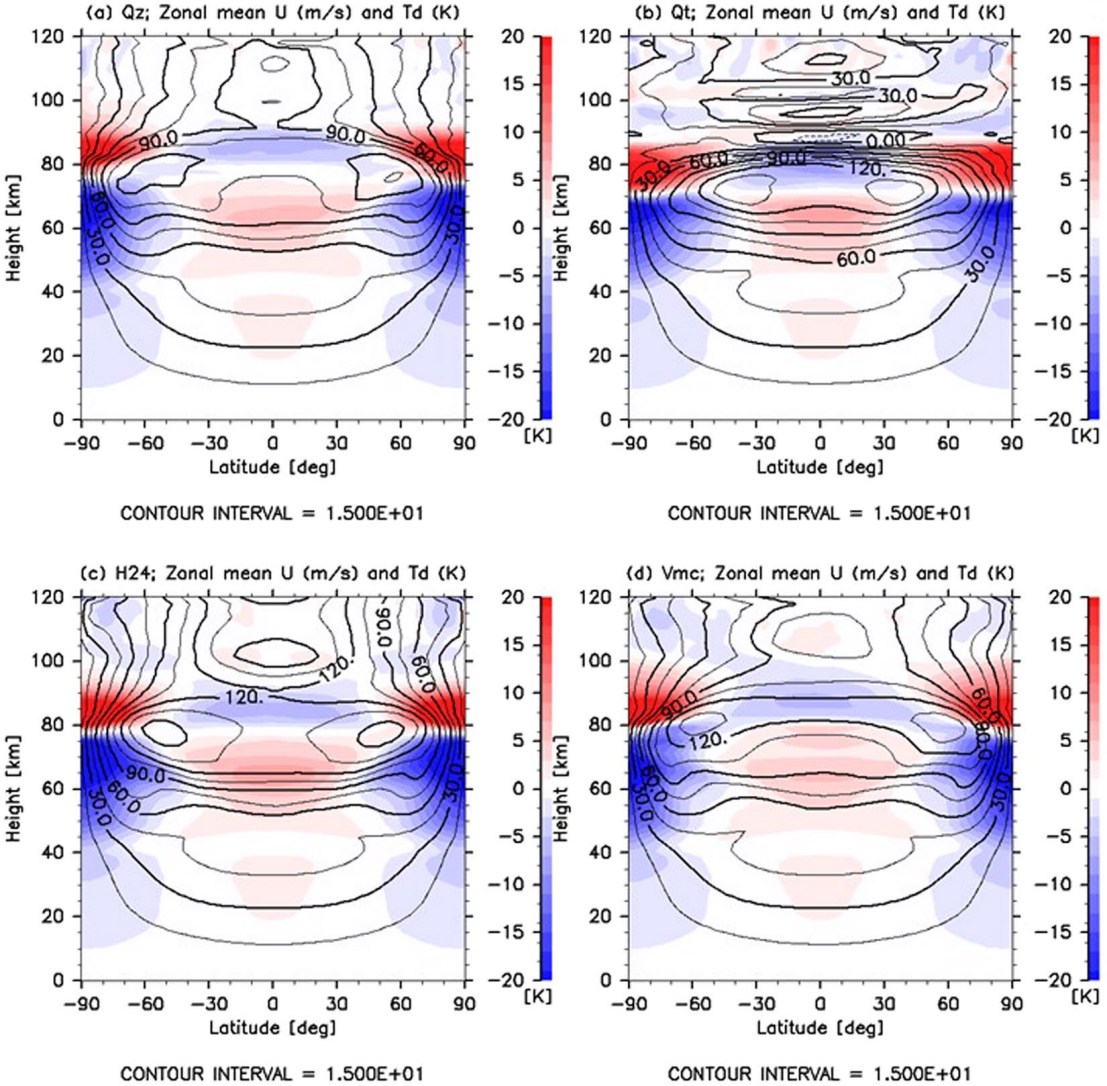
Latitude–height cross sections of zonal mean zonal wind ‘U’ (black contours; m s^−1^) and temperature deviation ‘${{\bf{T}}}_{{\bf{d}}}$’ from its horizontal average (colour shades; K) averaged over 20 Earth days for Cases (**a**) Qz, (**b**) Qt, (**c**) H24 and (**d**) Vmc.

Since the thermal tide propagates vertically, as shown in Figs 2 and 3, it is expected to transport zonal momentum upwards. Therefore, the general circulation may be substantially influenced in the upper layer of 70 km by data assimilation. Figure 4 shows latitude–height cross sections of the zonal mean zonal wind obtained in the quasi-equilibrium states for Cases Qz, Qt, H24 and Vmc. In Case Qz, without the thermal tide (Fig. 4a), strong mid-latitude jets caused by the enhanced mean meridional circulation (not shown) were found to emerge. In contrast, in Case Qt (Fig. 4b), the faster zonal wind at the equatorial region with mid-latitude jets shifted to the lower latitudes of 30°–45° appearing at the cloud-top level. In addition, in Case H24 (Fig. 4c), the faster zonal wind appeared at 60–90-km levels in low latitudes compared to that found in Case Qz. The meridional distribution of the zonal wind at the cloud level was intermediate as compared to those observed in Cases Qz and Qt. Furthermore, the fast zonal wind in low latitudes and the remarkable mid-latitude jets were similar to those found in Cases of Qt and Qz, respectively. In Case Vmc (Fig. 4d), while it seems that the zonal wind was somewhat accelerated at the cloud level, it was accompanied by remarkable mid-latitude jets, as found in Case Qz. It is worth noting that unlike Case Qt, the zonal wind in Cases H24 and Vmc were minimally decelerated above 75 km.

**Figure 5:**
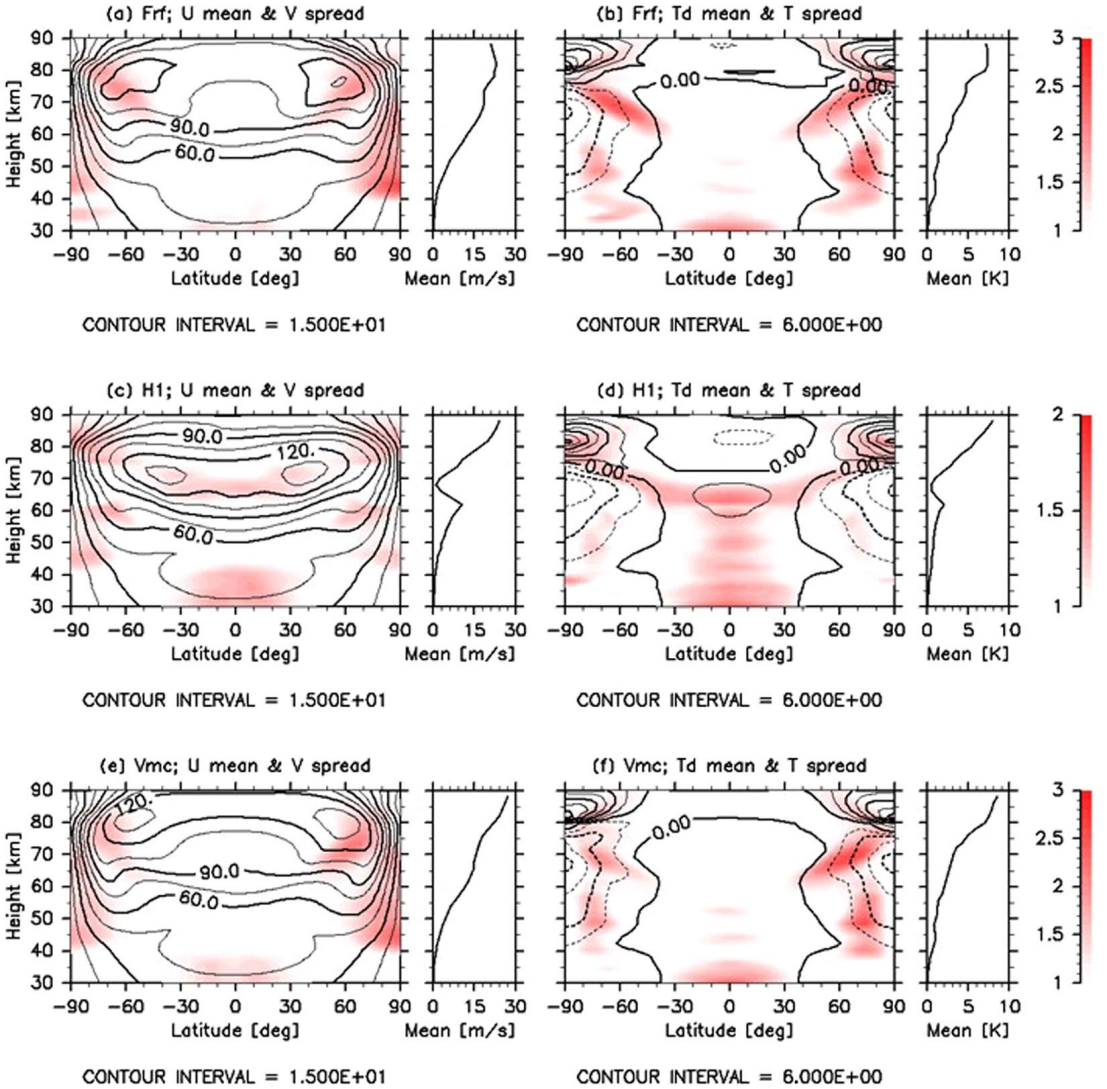
Latitude–height cross sections of the averaged ensemble spread in (**a**,**c** and **e**) meridional wind (colour shades; m s^−1^) and (**b**,**d** and **f**) temperature (colour shades; K) for Cases (**a**,**b**) Frf, (**c**,**d**) H1 and (**e**,**f**) Vmc. Values are normalised by their horizontal average at each altitude (line plots in right small panels). The zonal mean zonal winds ‘U’ (m s^−1^) and temperature deviations ‘${{\rm{T}}}_{{\rm{d}}}$’ (K) obtained from their horizontal average are also shown in black contours in panels (**a**,**c** and **e**) and (**b**,**d** and **f**), respectively. All values are averaged over 20 Earth days.

For a comprehensive observation, contours of the latitude–height cross sections of the zonal mean zonal wind obtained for Cases Frf, H1 and Vmc are shown in Fig. 5a,c and e, respectively. For Case Frf, without the thermal tide, strong mid-latitude jets were observed (Fig. 5a), which is similar to that in Case Qz (Fig. 4a). These were found to be common in previous GCM studies^22, 23^ that were conducted by excluding the thermal tide. In contrast, in Case H1, the faster zonal wind located in the equatorial region with mid-latitude jets shifted to the lower latitudes of 30°–45° and appeared at the cloud-top level (Fig. 5c), which is similar to that in Case Qt (Fig. 4b), as observed in other GCM studies^7, 8, 24, 25^ that were conducted considering the thermal tide. This meridional distribution of the superrotation also agrees well with the observations^26–28^. Furthermore, a remarkable deceleration of the zonal wind above 75 km was caused by the thermal tide^20^, which was also in good agreement with the zonal wind estimated from the observed temperature^29^. In Case Vmc (Fig. 5e), while the zonal wind was somewhat accelerated at the cloud level, it was accompanied by remarkable mid-latitude jets, as in Case Frf. Unlike Case H1, although the thermal tide was excited by data assimilation in Cases H24 and Vmc, the zonal wind was minimally decelerated above 75 km. For Case Vmc, approximately three-fourth of the horizontal area at 70 km did not have observational data, while all of the area investigated in Case H24 had observational data. Since we forced the VAFES run by Qz that includes only the zonal mean component, the atmospheric motions in Cases Vmc and H24 will ‘relax’ to those in Case Qz. This is largely due to the relatively sparse observations that were available within the approximate 24-h time intervals.

Because a strong latitudinal temperature gradient exists in the layer located at the 45–75-km level wherein the temperature difference between the equator and the pole is more than 25 K (Fig. 5b,d and f), baroclinic instability waves and Rossby-type waves^8, 25^ appeared in a weakly stratified layer located at 50–60 km. The ensemble spreads of the meridional wind and temperature for Cases Frf, H1 and Vmc are shown in Fig. 5 (colour). They were normalised by the horizontal average at each level (indicated by line plots in right small panels) in order to observe the latitudinal distributions. The spread indicates the extent to which the analysis can be trusted and the locations where disturbances actively appear. The horizontally averaged spreads increased with altitude due to the lower atmospheric density in the upper layer, except in Cases H1 and Vmc. In Cases H1 and Vmc, the spreads were significantly and slightly reduced, respectively, at approximately 70 km because the observations were limited to 70 km. Hence, this result suggests that the impacts of data assimilation extend over approximately 10 km in the vertical direction.

In Cases Frf and Vmc, the meridional distributions of the averaged spread showed that active disturbances appeared in two regions. One was located at the mid-latitudes of approximately 60°N (60°S) and extended from 60 to 80 km. In this area, the vertical shear and the latitudinal temperature gradient were significant. It has been inferred from previous research^8, 25^ that the large spreads could be caused by baroclinic instability waves. The other was located at high latitudes near the poles from 40 to 70 km (Fig. 5a and e). Since the vertical shear is small and the meridional gradient of the absolute vorticity changes its sign in this region (not shown), the large spreads could be caused by barotropic instability waves. In Case H1, similar structures can be observed in the spreads of meridional wind and temperature. The large spreads in the meridional wind also appeared at low latitudes from 60 to 70 km, and significant spreads in the temperature extended from 70 km to approximately 30 km. Since the thermal tide was excited at 70 km propagates downwards as well as upwards, these differences amongst the cases could be attributed to the structure of the zonal mean zonal wind affected by the thermal tide.

## Summary and Discussion

In this study, we developed a data assimilation system comprising the VAFES and the LETKF and applied it to the Venusian atmosphere for the first time. Since Venus is far from Earth and observational methods are quite restricted, detailed observational data, such as frequent multilevel winds and temperature, cannot be obtained as easily as they can be obtained for the terrestrial atmosphere. However, the results of this study confirmed that even the limited data acquired from satellite observations could significantly improve Venus GCM forecasts. Data assimilation using horizontal winds at a single altitude on the dayside corrected long period disturbances, such as those caused by the thermal tide.

It is strongly expected that the VAFES-LETKF analysis data produced from past and/or future observations will enable us to investigate and reconsider many important atmospheric features, such as the superrotation, the cold collar and the polar vortex. In addition, it was noted that the LETKF can be easily applied to any type of GCM. For example, the Laboratorie Meteorologie Dynamic (LMD) GCM^24, 25^ is one of the most advanced Venus GCMs that includes detailed physical processes. The LETKF could improve the physical parameters used in GCMs.

The Venus Climate Orbiter ‘Akatsuki’ began the observation of the Venusian atmosphere on 9 December 2015^30, 31^. This orbiter provides frequent data approximately every 2 h, which comprises cloud distributions, horizontal winds derived at multiple altitudes and temperature distributions at the cloud top^32^. The actual dynamics of atmospheric circulation on Venus remains unclear. Currently, there are many uncertainties, including baroclinic and/or barotropic instability waves, planetary waves, gravity waves and turbulences. We do not know their level of importance in Venusian atmospheric dynamics and their importance with regard to GCMs. It is strongly expected that the Akatsuki data with the VAFES-LETKF data assimilation system will enable us to reproduce more reliable models of the Venus atmosphere. Such reanalysis data will greatly help us to elucidate the actual atmospheric circulation and understand the dynamics of the Venusian atmosphere.

## Methods

We used a full nonlinear Venus GCM, named AFES-Venus (hereafter, VAFES)^7^, with simplified physical processes. This system is based on the AFES^6^. The resolution was set to T42L60, where T and L denote the triangular truncation number for spherical harmonics and the number of vertical levels, respectively. Then, there are 128 times 64 horizontal grids with 60 vertical levels. The vertical domain extended from the flat ground to approximately 120 km, with an almost constant altitude grid spacing of 2 km. The model included vertical and horizontal eddy diffusion. The vertical eddy diffusion coefficient was 0.15 m^2^ s^−1^. The horizontal eddy viscosity was represented by the second-order hyperviscosity. Damping time for the maximum wave number component was set at approximately 0.1 Earth days. Rayleigh friction with a damping time of 0.5 days was employed only at the lowest level to mimic the surface friction. In the upper atmosphere above 80 km, a sponge layer was assumed only for the eddy components and the damping times were gradually increased with height. A typical example of the increase in the damping time was 2500, 0.1 and 0.05 days, at 90, 100 and 110 km, respectively. Convective adjustment was also applied to eliminate static instability.

The vertical and horizontal distributions for solar heating were based on the research of Tomasko *et al*.^33^ Solar heating was decomposed into a zonal mean component and a deviation from the zonal mean (diurnal component), which excite the mean meridional (Hadley) circulation and the thermal tide, respectively. Two cases were simulated: Case Qt included both components, whereas Case Qz included only the zonal mean component. The infrared radiative process was simplified by a Newtonian cooling approximation wherein the coefficients of cooling were based on Crisp^34^. The relaxation time decreased almost exponentially from the surface to 120 km in approximately 25000–0.1 days (refer to Fig. 1a in Sugimoto *et al*.^7^). The temperature was relaxed to a prescribed horizontally uniform temperature distribution based on the Venus International Reference Atmosphere^35^. While the temperature was relaxed to the horizontally uniform field, the latitudinal gradient of the temperature was maintained by solar heating in this model. Further, the atmospheric motions, such as the Hadley cell and baroclinic instability waves, were driven by solar heating. Other details of the model settings were described in our previous research^7–9^.

The initial state of the velocity field was assumed to be an idealised superrotating flow in the solid-body rotation. The zonal wind increased linearly with height from the ground to 70 km. The velocity at the equator was set at 100 m s^−1^ at 70 km and was maintained constant above 70 km. Thus, the latitudinal profile of the initial zonal velocity above 70 km could be calculated by 100 × cos *θ* m s^**−**1^, where $\theta$ represents the latitude. The temperature distribution was set to be in gradient wind balance with the zonal wind to suppress the initial instability. It was assumed that the direction of the planetary rotation and the basic zonal wind was eastwards (positive). Using this initial state, we performed nonlinear numerical simulations for more than four Earth years in Cases Qt and Qz. The leapfrog method was employed for time integrations with increments of 600 s. The quasi-equilibrium datasets sampled at 1-h intervals in Case Qt were used as idealised observations, whereas those sampled at 8-h intervals in Case Qz were used as the initial conditions for each 31-member ensemble, which was used as the ensemble run in the data assimilation.

The local ensemble transform Kalman filter (LETKF) was based on previous research^2, 10–13^. It is an approximation of the Kalman filter and finds the best estimate (analysis) with minimum error variance in model estimates and in observations. Moreover, the LETKF is a square root filter method^36^ of the ensemble Kalman Filter^37^ and a deterministic filter in which no randomly perturbed observations are used. It is localised by considering only the observations within a prescribed horizontal and vertical distance^38, 39^. The ensemble transform Kalman filter^40^ approach is also used for acceleration. These techniques contribute to computational efficiency, and calculations are performed on massive parallel computers to produce a realistic high-dimensional atmospheric forecast model^41^. In the current VAFES-LETKF data assimilation system, the 31-member ensemble and 10% multiplicative spread inflation were employed. The localisation parameters were chosen to be 400 km in horizontal and log *P* = 0.4 in vertical, where *P* is pressure. We set observation errors with a 4.0-m s^−1^ standard deviation to the horizontal winds field, which was slightly less than the upper limit of the standard deviation of 7.0 m s^−1^ suggested by Kouyama *et al*.^15^. We checked the dependency of the results on these localisation parameters and observation errors and found that when these parameters or errors were set at double or half values, no significant changes were observed. Furthermore, a test case with a 63-member ensemble was also considered to check the saturation of the ensemble. The results indicated that the uncertainty of the model forecast was sufficiently characterized by the 31-member ensemble of the VAFES run. The time interval of the data assimilation cycle was set to 6 h. The four-dimensional LETKF comprised 7-h time slots at each analysis, and the observations were assimilated every hour depending on their availability^1, 21^.
